
JOURNAL INFORMATION
==============================
NLM Title Abbreviation: Antimicrob Resist Infect Control
Journal ID: Antimicrob Resist Infect Control
NLM Journal ID: 101585411

Antimicrobial Resistance and Infection Control

EISSN: 2047-2994

ARTICLE INFORMATION
==============================
PMCID: PMC8412969
DOI: 10.1186/s13756-021-00974-z
Article ID: 974
Article version: 1
Subjects: Meeting Abstracts

Abstracts from the 6th international conference on prevention & infection control (ICPIC 2021)

Electronic publication date: 2021 Sep 3
Publication date: 2021
Volume: 10
Issue: Suppl 1
Issue sponsor: Publication of this supplement was funded by ICPIC.
Electronic Location ID: 130
Copyright: © The Author(s) 2021
License: Open AccessThis article is licensed under a Creative Commons Attribution 4.0 International License, which permits use, sharing, adaptation, distribution and reproduction in any medium or format, as long as you give appropriate credit to the original author(s) and the source, provide a link to the Creative Commons licence, and indicate if changes were made. The images or other third party material in this article are included in the article's Creative Commons licence, unless indicated otherwise in a credit line to the material. If material is not included in the article's Creative Commons licence and your intended use is not permitted by statutory regulation or exceeds the permitted use, you will need to obtain permission directly from the copyright holder. To view a copy of this licence, visit http://creativecommons.org/licenses/by/4.0/. The Creative Commons Public Domain Dedication waiver (http://creativecommons.org/publicdomain/zero/1.0/) applies to the data made available in this article, unless otherwise stated in a credit line to the data.
License URL: https://creativecommons.org/licenses/by/4.0/

Conference name: International Conference on Prevention & Infection Control
Conference Acronym: ICPIC 2021
Conference location: Geneva, Switzerland
Conference date: 14-17 September 2021
issue-copyright-statement: © The Author(s) 2021

==============================
Publisher's Note

Springer Nature remains neutral with regard to jurisdictional claims in published maps and institutional affiliations.

